# PROTOCOL: PrEP (Non)Adherence Among Men Who Have Sex With Men: An Overview of Reviews: A Systematic Review

**DOI:** 10.1002/cl2.70047

**Published:** 2025-07-07

**Authors:** Guilherme G. Pinheiro, Carla Moleiro, David L. Rodrigues

**Affiliations:** ^1^ CIS‐Iscte Lisbon Portugal

## Abstract

This is the protocol for a Campbell systematic review. The objectives are as follows. The proposed overview of reviews aims to: (1) aggregate and systematize findings from multiple overviews of reviews, and offer a broader understanding of the factors, both facilitators and barriers related to PrEP awareness, uptake, and adherence among MSM; and (2) understand if there are gaps in the literature on potential facilitators and barriers that need greater analysis. Hence, the purpose of this overview of reviews aligns with Sustainable Development Goal 3 of the 2030 Agenda for Sustainable Development, particularly with SDG 3.3, that is, end the epidemics of AIDS and other communicable diseases.

## 1 Background

The human immunodeficiency virus (HIV) attacks the body's immune system by destroying a type of white blood cell (i.e., CD4 T lymphocyte) that helps the body deal with the infection. If not treated, HIV can lead to acquired immunodeficiency syndrome (AIDS) due to a high viral load (Deeks et al. 2015).

The introduction of antiretroviral therapy (ART) contributed to a sharp decline in death rates attributed to HIV during the 1990s (Mustanski et al. 2024). With innovations in the field of ART (e.g., antiretrovirals with lower levels of toxicity; Deeks et al. 2015), individuals living with HIV can have a similar lifespan compared to individuals who do not live with HIV (Finkelstein‐Fox et al. 2020; Mustanski et al. 2024).

Regardless of the innovations in HIV treatment and prevention, especially with the introduction of pre‐exposure prophylaxis (PrEP), the rates of new diagnoses have declined only moderately (Mustanski et al. 2024). This can be attributed to different factors, from inconsistent use of condoms (Mirandola et al. 2018; Shen et al. 2022) to low PrEP adherence (Halkitis et al. 2018; Meireles et al. 2020). Focusing on the latter, literature has been identifying factors that can impact PrEP use.

Low knowledge about PrEP, even among key population groups such as men who have sex with men (MSM) (Simões et al. 2021), fear about PrEP effectiveness and secondary effects (Halkitis et al. 2018), social representation of PrEP users (Spieldenner 2016), or a low perception of risk for HIV (Biello et al. 2018) have been identified as barriers to PrEP use. Nevertheless, PrEP use can empower individuals in their sexual lives and lead to a less negative perception of HIV (Mabire et al. 2019; Rojas Castro et al. 2019; Van Dijk et al. 2021). Indeed, PrEP can help reduce the fear associated with HIV and increase sexual satisfaction and sexual freedom (Van Dijk et al. 2021). Therefore, researchers should strive to understand how to foster adherence to new prophylaxis methods, including PrEP, particularly due to its cost‐effectiveness when compared to HIV treatment (Schackman et al. 2015).

Several reviews have already summarized empirical evidence on the facilitators and barriers connected to PrEP use among MSM. However, some of the reviews target only a subgroup within MSM (e.g., black MSM; Russ et al. 2021), use a specific methodology (e.g., qualitative research; Ching et al. 2020), or are restricted to a certain geographic location (e.g., USA context; Dang et al. 2022). As such, the existing reviews fail to provide a comprehensive view of the aspects related to PrEP use among MSM, leading to the need for an overview or reviews.

We propose using the PrEP care continuum as the organizing structure in our review of the facilitators and barriers. This framework was proposed by Nunn et al. (2017) and was initially used to assess progress in PrEP implementation across a continuum, as PrEP‐related outcomes differ from the HIV treatment continuum. Researchers adopted it to understand the factors leading to each stage (e.g., Russ et al. 2021). The framework defines the stage at which people may be regarding PrEP, starting with awareness, moving to uptake, and then to adherence and retention (Nunn et al. 2017). The awareness stage involves identifying individuals at higher risk of HIV and raising their perception of risk to promote PrEP. This includes improving knowledge about PrEP among population groups to address misconceptions and gaps in awareness. Uptake covers the steps necessary for individuals to access PrEP care. Adherence focuses on ensuring that individuals take PrEP consistently as prescribed and remain engaged in PrEP care, which is critical for PrEP's efficacy in preventing HIV. Finally, retention ensures monitoring, support, and adjustments as needed (Nunn et al. 2017).

A search for registered overviews of reviews was conducted on PROSPERO, OSF, Campbell, Cochrane, JBI, PubMed, and Scopus. A total of four overviews of reviews were identified. On PROSPERO, the search revealed the existence of two ongoing overviews of reviews, one focusing on multiple populations (Jin et al. 2024; CRD42023421747), and another on MSM (Gaetani et al. 2024; CRD42024577519). A search on PubMed showed two published overviews of reviews, one focusing on multiple populations (Jin et al. 2023), and the other focusing on the context of the United States (Dhir 2023). A Scopus search revealed two results, both previously identified on PubMed (Dhir 2023; Jin et al. 2023). The search on Campbell, Cochrane, JBI, and OSF did not return results of overviews of reviews targeting PrEP among MSM, apart from scoping, systematic reviews, and meta‐analyses, which are different types of reviews.

Unlike the identified overviews of reviews that considered multiple populations, our review focuses exclusively on MSM and considers the multiple intersecting identities within this population, which we expect will lead to a deeper understanding of the experienced facilitators and barriers along the PrEP care continuum. We broaden the scope by including both English and non‐English publications, and gray literature to ensure a more comprehensive synthesis of the available evidence, and to differentiate our work from existing overviews targeting MSM, multiple populations, or focusing on a specific context.

We acknowledge the importance of understanding an individual's experience of belonging to one social group in relation to their belongingness to other social groups (McCormick‐Huhn et al. 2019). We have chosen to focus on MSM, defining men as any individual who self‐identifies as such. This allows us to synthesize and systematize findings from multiple reviews and provide a broader understanding of the topic by considering the nuances that intersectionality can add in terms of facilitators and barriers for MSM across the PrEP care continuum.

## 2 Objectives

The proposed overview of reviews aims to: (1) aggregate and systematize findings from multiple overviews of reviews, and offer a broader understanding of the factors, both facilitators and barriers related to PrEP awareness, uptake, and adherence among MSM; and (2) understand if there are gaps in the literature on potential facilitators and barriers that need greater analysis. Hence, the purpose of this overview of reviews aligns with Sustainable Development Goal (SDG) 3 of the 2030 Agenda for Sustainable Development, particularly with SDG 3.3, that is, end the epidemics of AIDS and other communicable diseases.

## 3 Methods

This overview of reviews will be conducted under the JBI methodology for umbrella reviews (Aromataris et al. 2015, 2024). To ensure a transparent, complete, and accurate account of the review process, an updated Preferred Reporting Items for Systematic Reviews and Meta‐Analyses (PRISMA) reporting guidelines (Page et al. 2021) will be used.

### 3.1 Eligibility/Inclusion Criteria

#### 3.1.1 Participants

This overview of reviews will include reviews that focus on MSM, regardless of their relationship status. We considered MSM individuals who identify themselves as men (i.e., individuals who were assigned female at birth but identify as men, trans men; and individuals assigned male at birth who identify as men, cis men). Reviews targeting underage MSM or MSM living with HIV will not be considered. Moreover, reviews targeting mixed populations will only be included if the review findings related to MSM can be extracted and if data have not been captured in an already included review to avoid data duplication.

#### 3.1.2 Concept

This overview of reviews will consider reviews that report on factors regarding oral PrEP use (i.e., facilitators and barriers) at any stage of the PrEP care continuum (Nunn et al. 2017). We define facilitators as factors that can be promoted to increase positive health‐related outcomes, and barriers as factors that should be mitigated to prevent negative health‐related outcomes. Reviews targeting other forms of PrEP (e.g., injectable) will not be considered.

#### 3.1.3 Context

This overview of reviews will have no restrictions regarding geographical location, cultural and/or socioeconomic context.

#### 3.1.4 Types of Sources

This overview of reviews will consider systematic reviews, scoping reviews, meta‐analyses, and narrative reviews of quantitative, qualitative, and mixed methods if (a) clearly defined review question(s) and eligibility criteria to select primary studies are provided, and (b) if the strategy of the search and selection process that includes at least one bibliographic database is described in detail.

### 3.2 Search Strategy

The search strategy will aim to locate published and unpublished reviews of quantitative, qualitative, and mixed‐method studies.

Following JBI recommendations for umbrella reviews (Aromataris et al. 2015, 2024), a three‐step search strategy will be conducted to define a draft set of search terms and search strings. First, we will perform an initial limited search of Academic Search Complete, APA PsycArticles, APA PsycInfo, E‐Journals, Psychology and Behavioral Sciences Collection (all via EBSCO), and PubMed to identify articles on the topic. Then, the text contained in the title, abstract, and index terms used to describe these articles will be analyzed. This will allow the identification of the text words and index terms, including MeSH terms, that match the inclusion criteria of this overview of reviews.

Aiming to provide complete and relevant multidisciplinary coverage, the final databases will be from the areas of medical and social sciences, namely, Cochrane Library, JBI Evidence Synthesis, PubMed, Scopus, Web of Science, Global Index Medicus, and SciELO.

To capture relevant gray literature, we will include all non‐peer‐reviewed papers (e.g., dissertations) retrieved from the final databases (e.g., Web of Science includes ProQuest Dissertations & Theses Citation Index). Hence, reducing the risk of publication bias and identifying as much relevant evidence as possible.

Search terms will be developed around four key themes: (i) MSM; (ii) pre‐exposure prophylaxis; (iii) barriers/facilitators; (iv) review. As for search strings, terms belonging to the same theme will be combined with OR, and terms belonging to different themes will be combined with AND. Search strings will be finalized and customized for each included database and information source. Team members agreed on the draft search strategy, which was analyzed by an information specialist for validation and refinement purposes. A detailed example of the proposed search terms and search strings can be found in Appendix SI.

The search strategy will include date and language restrictions. We will include reviews from 2012 onwards. This year was chosen because it was the year in which the use of antiretrovirals as PrEP was first approved. Regarding language, based on the research team's knowledge, we will consider English, Portuguese, and Spanish papers to access literature on non‐Anglophone countries. Reviews published in languages other than the ones mentioned or before the selected date will be excluded.

#### 3.2.1 Study Selection

Following the search, all identified citations will be collated and uploaded into Rayyan, a research collaboration platform designed to support the conducting of literature reviews in terms of records organization and management (Ouzzani et al. 2016). Duplicates will then be removed, followed by a pilot test with 20 records to verify the clarity of the eligibility criteria and to refine the screening strategy. After the pilot test, titles and abstracts will be screened for compliance with the review inclusion criteria by two independent reviewers. Any disagreements will be resolved through discussion between the two reviewers or with the help of a third reviewer. If uncertainty or disagreement persists, the record will be included in the full‐text screening phase. This will help us reduce the risk of bias during the selection process.

In the next step, potentially relevant records will be retrieved in full and assessed against the review inclusion criteria, using a detailed assessment guide. The assessment process will be conducted by two independent reviewers. Any disagreements will again be resolved through discussion between review team members until a consensus is reached. Full‐text records excluded from the review for not meeting the eligibility criteria, as well as the reasons for their exclusion, will be documented in the final report of this overview of reviews.

The final report will present the results of the search and selection processes in full, through the narrative and the PRISMA 2020 flow diagram for systematic reviews (Page et al. 2021).

#### 3.2.2 Assessment of Methodological Quality

Methodological quality will be assessed following JBI recommendations for umbrella reviews (Aromataris et al. 2015, 2024). Therefore, critical appraisal will be assessed by two independent reviewers using JBI's checklist for systematic reviews and research syntheses. This checklist consists of 11 items assessing the adequacy of the review process and the clarity of its reporting. For each item, there will be four response options, including “yes,” “no,” “unclear,” and “not applicable.” Any disagreements that arise between the reviewers will be resolved through discussion. If necessary, assistance from a third reviewer will be requested. A “yes” will be assigned when the information regarding an item is clearly and explicitly stated, a “no” when there is no mention of the information in the checklist parameter, and “unclear” if the review referred to, but did not clearly and explicitly state the information regarding an item. The appraisal results will then be classified as high quality (i.e., for reviews with ≥ 9 “yes” answers), moderate quality (i.e., for reviews with 6–8 “yes” answers), low quality (i.e., for reviews with 3 to 5 “yes” answers), and very low quality (i.e., for reviews with ≤ 2 “yes” answers). The results of the quality assessment will be presented in narrative and table format in the final report of the overview of reviews.

As this overview of reviews will consider different types of sources and with varying levels of methodological robustness (e.g., systematic reviews and scoping reviews), the results of the quality assessment will not be used for inclusion and exclusion purposes. Instead, they will be considered to guide interpretations of the overview of the review's findings and inform its strengths and weaknesses.

#### 3.2.3 Data Extraction

Data from the included reviews will be extracted by two independent reviewers using a data extraction tool developed by the review team members. Data extracted will include details describing each review, such as type of review, review aim(s), PrEP care continuum stage, and key findings. In cases where additional data is needed, the authors of the reviews will be contacted to provide all necessary information. Plus, we will ensure that there are errata regarding each included study.

Recognizing that not all included reviews will consider the PrEP care continuum, we anticipate the need to categorize the data. Therefore, two researchers will independently code the reviews (e.g., reviews focusing on factors related to willingness to use PrEP will be coded in the uptake stage, considering that uptake requires an individual to already be aware of PrEP), and a third member of the research team will help resolve any remaining uncertainties or disagreements.

A draft data extraction tool can be found in Appendix SII. To minimize the risk of errors in data extraction, the strategy will first be discussed among the review team members and tested through a pilot test of five reviews. If necessary, the draft data extraction tool will be modified and revised. Modifications will be detailed in the final report of the overview of reviews. Any disagreement between the reviewers will be solved through discussion. If consensus cannot be reached, a third reviewer will be consulted.

#### 3.2.4 Data Summary

Data obtained from the included reviews will be organized into tables and accompanied by a narrative to address the review questions in line with the inclusion criteria. All results will be subject to double data entry. Data from quantitative, qualitative, and mixed‐method studies will be presented separately, being organized according to the PrEP care continuum regarding the facilitators and barriers identified. After being summarized, the quantitative and qualitative data will be analyzed, critically compared, and then discussed in terms of their convergence, complementarity, or divergence.

Findings of the overview of reviews will be presented in a “Summary of Findings” table along with the quality evaluation rates given based on methodological limitations of the included overview of reviews, as well as their consistency, risk of bias, and relevance to the population of interest.

## Author Contributions

- Content: Guilherme G. Pinheiro.
- Review methods: Carla Moleiro and David L. Rodrigues.
- Information retrieval: Guilherme G. Pinheiro.

## Conflicts of Interest

The authors declare no conflicts of interest.

## Preliminary Timeframe

Date planned to submit a draft review: 31/04/2026.

## Plans for Updating This Review

We aim to complete the overview of reviews within 1 year after the final database search. If this timeline is not met, we will perform an updated search to include studies published after the initial search.

## Supporting information

Appendix.
