# Supplementary material for: PROTOCOL: PrEP (Non)Adherence Among Men Who Have Sex With Men: An Overview of Reviews: A Systematic Review
Source: Campbell Syst Rev. 2025 Jul 7;21(3):e70047. doi: 10.1002/cl2.70047 (PMC12230767; doi:10.1002/cl2.70047)
Supplement: Supplementary file 1 — Appendix. [file CL2-21-e70047-s001.docx]

# Appendices

##### Appendices should be submitted as [supplementary material](https://authorservices.wiley.com/author-resources/Journal-Authors/Prepare/manuscript-preparation-guidelines.html/supporting-information.html).

## 1 Example - Search strategies:

PUBMED

| # | Search |
| --- | --- |
| 1 | [Mesh terms] "Sexual and Gender Minorities" OR "Homosexuality, Male" OR "Transgender Persons" |
| 2 | TI/AB (“men who have sex with men” OR MSM OR male* OR trans* OR “high risk group*” OR “high-risk group*” OR “key population*”) |
| 3 | [Mesh terms] "Pre-Exposure Prophylaxis" |
| 4 | TI/AB (“pre-exposure prophylaxis” OR “pre exposure prophylaxis” OR “preexposure prophylaxis” OR “HIV prophylaxis” OR “antiretroviral prophylaxis”) |
| 5 | [Mesh terms] "Motivation" OR “Attitude" OR "Intention" OR "Health Knowledge, Attitudes, Practice" OR "Patient Acceptance of Health Care" OR "Medication Adherence" OR "Treatment Adherence and Compliance" |
| 6 | TI/AB (adherence OR uptake OR awareness OR motivation* OR attitud*) |
| 7 | TI/AB (review OR “meta analysis” OR “meta-analysis” OR “meta synthesis” OR “meta-synthesis” OR metasynthesis OR “evidence-based synthesis” OR “evidence-based analysis” OR "umbrella review" OR "overview of reviews" OR "review of reviews") |
| 8 | (#1 OR #2) AND (#3 OR #4) AND (#5 OR #5) AND #7 |

## 2 Data extraction instrument

| **Review details** |
| --- |
| *Author* |
| *Year of publication* |
| *Type of review* |
| *Review goal(s)* |
| *Participants characteristics* |
| *Geographical location* |
| **Search strategy** |
| *Sources searched* |
| *Publication year range of included reviews* |
| *Number of studies included* |
| *Type of studies included* |
| **Appraisal** |
| *Critical appraisal instrument used* |
| *Critical appraisal rating or interpretation* |
| **Relevant findings to the present review of reviews**  **- Quantitative reviews**  PrEP care continuum stage  Factor(s) reported by the review  **- Qualitative reviews**  PrEP care continuum stage  Factor(s) reported by the review  **- Mixed methods reviews**  PrEP care continuum stage  Factor(s) reported by the review |
| **Comments** |
